# Innate Immunity and Platelets: Unveiling Their Role in Chronic Pancreatitis and Pancreatic Cancer

**DOI:** 10.3390/cancers17101689

**Published:** 2025-05-17

**Authors:** Juliane Blümke, Moritz Schameitat, Atul Verma, Celina Limbecker, Elise Arlt, Sonja M. Kessler, Heike Kielstein, Sebastian Krug, Ivonne Bazwinsky-Wutschke, Monika Haemmerle

**Affiliations:** 1 Institute of Pathology, Section of Experimental Pathology, Medical Faculty, Martin Luther University Halle-Wittenberg, 06112 Halle (Saale), Germany; juliane.bluemke@uk-halle.de; 2 Institute of Anatomy and Cell Biology, Medical Faculty, Martin Luther University Halle-Wittenberg, 06108 Halle (Saale), Germany; moritz.schameitat@uk-halle.de (M.S.); celina.limbecker@uk-halle.de (C.L.); heike.kielstein@uk-halle.de (H.K.); ivonne.bazwinsky@uk-halle.de (I.B.-W.); 3 Department of Internal Medicine I, Medical Faculty, Martin Luther University Halle-Wittenberg, 06120 Halle (Saale), Germany; atul.verma@uk-halle.de (A.V.); sebastian.krug@med.uni-heidelberg.de (S.K.); 4 Institute of Pharmacy, Experimental Pharmacology for Natural Sciences, Faculty of Natural Sciences, Martin Luther University Halle-Wittenberg, 06120 Halle (Saale), Germany; sonja.kessler@pharmazie.uni-halle.de; 5 Department of Internal Medicine IV, University Hospital Heidelberg, 69120 Heidelberg, Germany

**Keywords:** innate immune cells, immune microenvironment, platelets, pancreatitis, PDAC

## Abstract

Pancreatic ductal adenocarcinoma (PDAC) is one of the most aggressive and lethal forms of cancer, characterized by a highly desmoplastic tumor microenvironment. One main risk factor is chronic pancreatitis (CP). Progression of CP to PDAC is greatly influenced by persistent inflammation promoting genomic instability, acinar–ductal metaplasia, and pancreatic intraepithelial neoplasia (PanIN) formation. Components of the extracellular matrix, including immune cells, can modulate this progression phase. This includes cells of the innate immune system, such as natural killer (NK) cells, macrophages, dendritic cells, mast cells, neutrophils, and myeloid-derived suppressor cells (MDSCs), either promoting or inhibiting tumor growth. On one hand, innate immune cells can trigger inflammatory responses that support tumor progression by releasing cytokines and growth factors, fostering tumor cell proliferation, invasion, and metastasis. On the other hand, they can also activate immune surveillance mechanisms, which can limit tumor development. For example, NK cells are cytotoxic innate lymphoid cells that are able to kill tumor cells, and active dendritic cells are crucial for a functioning anti-tumor immune response. In contrast, mast cells and MDSCs rather support a pro-tumorigenic tumor microenvironment that is additionally sustained by platelets. Once thought to play a role in hemostasis only, platelets are now recognized as key players in inflammation and cancer progression. By releasing cytokines, growth factors, and pro-angiogenic mediators, platelets help shape an immunosuppressive microenvironment that promotes fibrotic remodeling, tumor initiation, progression, metastasis, and immune evasion. Neutrophils and macrophages exist in different functional subtypes that can both act pro- and anti-tumorigenic. Understanding the complex interactions between innate immune cells, platelets, and early precursor lesions, as well as PDAC cells, is crucial for developing new therapeutic approaches that can harness the immune and potentially also the coagulation system to target and eliminate tumors, offering hope for improved patient outcomes.

## Simple Summary

Chronic pancreatitis (CP) is a common risk factor for pancreatic ductal adenocarcinoma (PDAC), which is a highly aggressive cancer. The development of PDAC is influenced by interactions between tumor cells and their surrounding microenvironment, including immune cells as well as platelets. Immune cells can either help or hinder tumor growth, while platelets play a major role in supporting tumor growth and metastasis. Understanding these interactions is key to developing treatments that impact the immune and coagulation systems to fight PDAC and improve patient outcomes.

## 1. Introduction

**Figure 1:**
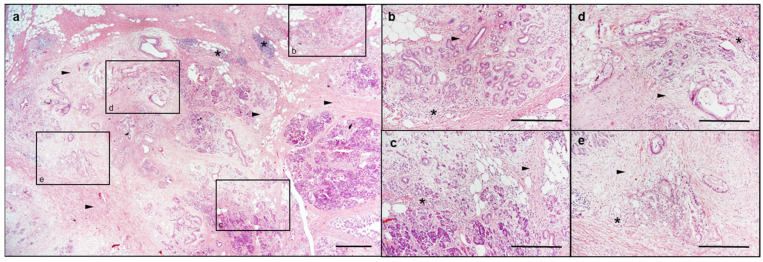
Histology of PDAC with associated features of chronic pancreatitis. Overview of an HE-stained section of a pancreatic resection specimen (**a**) of a patient with PDAC (**d**,**e**) with associated parenchyma atrophy and extensive inter- and intralobular fibrosis with interstitial inflammation and ADM lesions of surrounding peritumoral pancreatic tissue characterizing chronic pancreatitis (**b**,**c**). Arrowheads mark areas with fibrosis and/or desmoplasia, asterisks show areas of inflammation. Scale bar for (**a**) 1000 µm, scale bar for (**b**–**e**) 500 µm.

Chronic pancreatitis (CP) is a major cause of morbidity and a global burden for healthcare systems [1]. It is a chronic inflammatory condition of the pancreas leading to progressive scarring of pancreatic tissue, endocrine and exocrine tissue dysfunction, and increased risk for pancreatic cancer development [2,3,4]. Persistent tissue damage of the pancreas is caused primarily by prolonged alcohol abuse or smoking, and more rarely by hereditary genetic disorders [5,6,7]. This leads to direct or indirect cellular damages that result in an inflammatory reaction, activation of pancreatic stellate cells, and ultimately replacement of orthotopic pancreatic tissue with fibrotic tissue. The key histological features (Figure 1a–c) are intralobular, interlobular and periductal fibrosis, atrophy of acinar parenchyma and acinar–ductal metaplasia (ADM), ductal changes including ductal dilation and distortion with protein plugs and calcifications and associated interstitial inflammation [8,9], that ultimately persist after progression to pancreatic ductal adenocarcinoma (PDAC, Figure 1a,d,e).

It is well-recognized that CP is a risk factor for PDAC development. A large multicenter retrospective study found that subjects with chronic pancreatitis had a 14-fold increased risk of developing PDAC [4]. Other studies estimate the risk of PDAC development in patients with CP to be even higher [10]. Mechanistically, both genetic as well as epigenetic mechanisms, subsequent inflammatory processes, and oxidative stress resulting in DNA damage have been implicated in the progressive development of PDAC from CP [11]. A recent study aimed to identify hub genes in the development of CP and PDAC by analyzing transcriptomic data of various publicly available datasets. This study identified the gene *CEL* (carboxyl ester lipase), a glycoprotein secreted from the pancreas, as particularly important for CP and PDAC, and its expression correlated with the pancreatic immune cell landscape [12]. Others identified genes related to cell cycle and DNA replication to be key for the development of CP and pancreatic cancer [13]. In general, chronic inflammation, as seen in CP, is known to cause DNA damage, endoplasmic reticulum (ER) stress and autophagy. Tumor-promoting inflammation is one of the prerequisites for sustaining proliferative activity and promoting angiogenesis [14,15,16]. Finally, the accumulation of mutations in acinar or ductal cells of the pancreas can lead to dysplasia and the development of PDAC [17]. Based on the most recent global cancer statistics, PDAC is the third leading cause of cancer-related death worldwide [18]. The molecular pathology of PDAC is dominated by activating mutations in codon 12 of the proto-oncogene *KRAS* that occur in >90% of PDACs [19,20]. In the course of the disease, additional mutations arise, including inactivating mutations in tumor suppressor genes like *TP53*, *CDKN2A*, or *SMAD4*. It is well accepted that *KRAS* mutations occur very early in disease development and are already detected in preneoplastic lesions, including ADM and pancreatic intraepithelial neoplasia (PanIN) [21,22,23]. In mice, a *KRAS* G12D mutation is able to promote ADM and PanIN formation and spontaneously induce invasive cancer [24], accelerated by an additional inflammatory trigger [25,26]. Interestingly, *KRAS* mutations not only regulate intrinsic signaling pathways to sustain cellular proliferation and inhibit apoptosis in mutated cells. They are also able to regulate the tumor microenvironment (TME) by supporting a tumor-promoting inflammatory milieu that eventually contributes to immune escape [27]. In general, it is believed that KRAS induces several inflammatory cytokines and chemokines as well as signaling pathways, which promote tumorigenesis. For example, *KRAS* mutations have been shown to promote the progression of PanIN lesions and PDAC, which depended on the secretion of IL-6 (interleukin-6) by myeloid cells, activation of STAT3 (signal transducer and activator of transcription 3) and, SOCS3 (suppressor of cytokine signaling 3) [28]. In addition, KRAS and IL-6 synergism caused activation of the mitogen-activated protein kinase (MAPK) signaling pathway that was responsible for the initiation of pancreatic cancer precursor lesions [29]. Similarly to IL-6, the CXCR2 (C-X-C motif chemokine receptor 2)/IL-8 axis induced by KRAS was shown to be crucial for promoting phenotype alterations of CAFs (cancer-associated fibroblasts) and increased secretion of pro-tumoral cytokines that eventually promoted the development of PDAC [30]. Moreover, STAT5 activation downstream of oncogenic KRAS signaling can trigger inflammation-mediated ADM formation and PDAC growth [31]. Interestingly, oncogenic *KRAS^G12D^* can be transferred to tumor cells via platelet-like particles leading to a growth advantage in *KRAS* wildtype cells [32]. In addition, it was shown that *KRAS*-mutated cancer cells induce platelet aggregation [33], a phenomenon called TCIPA (tumor cell-induced platelet aggregation) that is associated with metastasis formation and might be an emerging therapeutic target in cancer therapy [34]. Once activated by tumor cells, platelets can secrete a plethora of inflammatory cytokines that lead to the recruitment and activation of immune cells, which then secrete chemotactic factors to regulate other immune cells [35]. Various classes of regulatory and cytotoxic immune cells were frequently identified within the microenvironment of the pancreas and pancreatic cancer tissue [36,37,38], showing differences in diverse tumor areas as well as changes upon disease progression and neoadjuvant therapy. During early cancer progression, immune cells are still able to eliminate precancerous cells. However, tumor cytotoxicity or anti-tumor immunity are mainly suppressed in PDAC, suggesting increasing dysfunction of the immune response, which is likely mediated by the PDAC cells themselves [35,39]. Moreover, tumor-associated immune cells can be divided into tumor-antagonizing and tumor-promoting immune cells, playing various roles in the different stages of tumor progression [40]. PDAC cells can influence the metabolic reprogramming of immune cells [41]. In addition, the interaction of immune cells with platelets, both in the circulation and within the TME, appears to play a critical role in modulating immune cell infiltration, differentiation, and activity, thereby regulating tumor growth and immune evasion [42,43,44]. Altogether, this suggests that both genetic and non-genetic factors regulate microenvironmental changes in pancreatic tissue that impact tumorigenesis and cancer dissemination. Moreover, persistent organ injury and chronic inflammation induce DNA damage in preneoplastic lesions, ultimately resulting in the breaching of basement membranes and invasive cancer.

In this review, we focus on the role of innate immune effector cells and platelets in chronic pancreatitis and pancreatic cancer, given their involvement in tissue injury and repair, fibrosis, stromal remodeling, tumor growth, and metastasis. We focused on the main key signaling pathways that drive the reorganization of the tumor immune microenvironment in PDAC, highlighting their potential as therapeutic targets. Additionally, we emphasize the emerging role of platelets as orchestrators of innate immunity and potential facilitators of immune evasion in cancer.

## 2. Innate Immune Cells in Chronic Pancreatitis and PDAC

**Figure 2:**
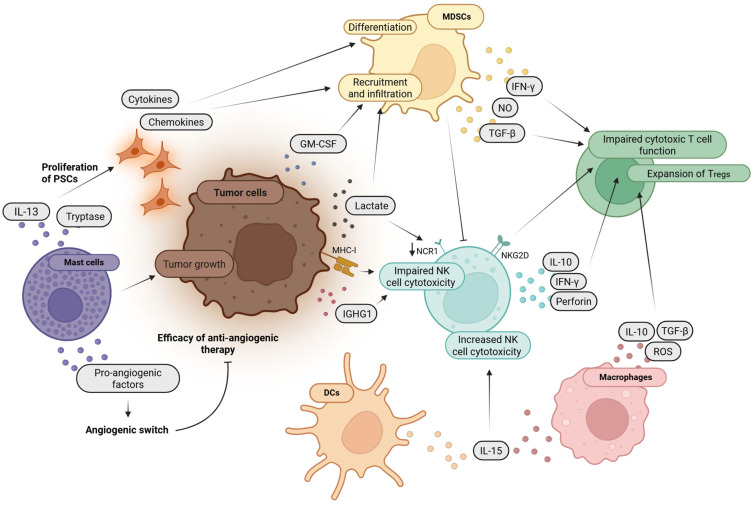
Interplay of tumor cells with NK (natural killer) cells, MDSCs (myeloid-derived suppressor cells) and mast cells. Tumor cell secretion of lactate and IGHG1 (Igγ-1 chain C region) leads to an impaired NK cell cytotoxicity, e.g., via downregulation of the surface receptor NCR1. Additionally, MHC (major histocompatibility complex) class I molecules on the tumor cell surface similarly suppress NK cell cytotoxicity. Increased NK cell cytotoxicity is mediated via IL-15 (interleukin-15), secreted by DCs (dendritic cells) or macrophages. Active NK cells can reduce cytotoxic T cell function and regulate the expansion of Tregs either directly by engaging NKG2D or indirectly by the secretion of IFN-γ (interferon gamma), IL-10 or perforin. Other mechanisms that can limit adaptive immune responses include secretion of cytokines by MDSCs or macrophages, including TGF-β (transforming growth factor beta) among others. MDSCs are recruited via tumor cell-derived lactate or GM-CSF (granulocyte-macrophage colony-stimulating factor). In addition, PSCs (pancreatic stellate stells) support the recruitment and infiltration as well as differentiation of MDSCs via the STAT3 (signal transducer and activator of transcription 3) pathway. Proliferation of PSCs is induced via mast cell-derived IL-13 and tryptase. In addition, mast cells are considered tumor-promoting in PDAC and secrete pro-angiogenic factors that induce an angiogenic switch and limit the efficacy of anti-angiogenic therapy. The figure was created with BioRender.com.

**Figure 3:**
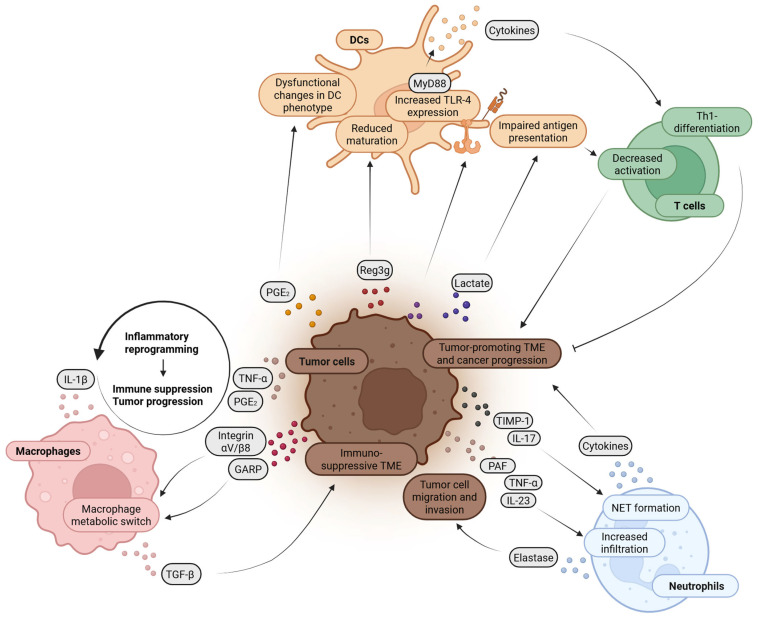
Interplay of tumor cells with macrophages, DCs and neutrophils. Integrin αV/β8 and GARP (glycoprotein a repetitions predominant) secreted by tumor cells lead to a metabolic switch in macrophages promoting the secreting of TGF-β that supports an immunosuppressive and tumor-promoting TME (tumor microenvironment). In addition, tumor cells secreted TNF-α (tumor necrosis factor alpha) and PGE_2_ (prostaglandin E_2_) are part of a positive feedback loop, which triggers IL-1β secretion by macrophages. This sustains a tumor-promoting inflammatory response, facilitating immune suppression and tumor progression. Tumor cell-derived PGE_2_, Reg3g (regenerating islet-derived protein 3 gamma) and lactate induced dysfunctional phenotypic changes in DCs, reduced DC maturation and impaired antigen presentation by DCs, respectively. In addition, tumor cells increase TLR4 (toll-like-receptor 4) expression in DCs which leads to a secretion of cytokines via the MyD88 pathways that favor Th1 cell differentiation. In summary, tumor cells can influence DCs in ways that induce both anti-tumorigenic and pro-tumorigenic effects in the T cell compartment. A close interaction also exists between tumor cells and neutrophils. Tumor-derived cytokines induce infiltration of neutrophils into tumor tissue and induce NET formation. On the other hand, neutrophils secrete cytokines that support a tumor-promoting TME and induce tumor cell migration and invasion. The figure was created with BioRender.com.

Innate immune cells are active players in the pathogenesis of both CP and PDAC. They perpetuate chronic inflammation, shape the tumor microenvironment, promote or inhibit tumor progression, and influence immune evasion. In addition, they are altered in their function, maturation, or number which might have clinical implications. Crucial functions and intercellular interactions of innate immune cells and tumor cells and their impact on T cells within the TME are summarized in Figure 2 and Figure 3 and described in more detail in the following paragraphs.

### 2.1. Natural Killer (NK) Cells

#### 2.1.1. Key Physiological Functions

Natural killer (NK) cells have been the focus of fundamental research for quite some time, as they represent an efficient endogenous first-line defense against cancer [45,46]. As part of the innate immune system, NK cells are “naturally” cytotoxic, which means they are able to identify virus-infected [45] or malignant cells and eliminate them via induction of apoptosis without prior antigen exposure. The activity of NK cells is balanced by activating and inhibitory receptors on their surface [47,48,49,50,51], which in turn are affected by activating and inhibitory signals. NK cells recognize if a proximal cell expresses a ligand profile associated with oncogenic transformation. The joint activating and inhibitory signals decide whether a cell is targeted for killing [52]. Activating NK cell receptors recognize stress-induced ligands on the target cells, as well as other alert molecules and toll-like-receptor ligands. Additionally, NK cells are able to exert ADCC (antibody-dependent cell cytotoxicity) by recognizing antibody-coated target cells with the Fc receptor CD16 [53]. Inhibitory receptors recognize constitutively expressed self-molecules on potentially altered target cells and induce NK cell tolerance, especially the MHC-I (major histocompatibility complex class I molecule) [54]. According to the “missing self”-hypothesis, NK cells contribute to T cell-mediated killing by eliminating cells that lack MHC-I [50]. However, other inhibitory ways exist that are independent of MHC-I molecules. In this context, the inhibition of NK cells via the 2B4 receptor is one of the best-characterized mechanisms. Other NK-cell inhibitory receptors that have non-MHC-molecule ligands include, for example, the NK-cell-receptor protein 1 family, the carcinoembryonic antigen family, the sialic-acid-binding immunoglobulin-like lectins [55].

#### 2.1.2. Pathophysiology in PDAC

NK cells should kill single tumor cells before they develop into solid tumors. Also, they should contribute to preventing metastasis by eliminating circulating tumor cells [56]. Previous studies have shown that the pathophysiology and progression of pancreatic cancer are strongly affected by the interaction of cancer cells with the TME, and are closely associated with dysfunctional NK cells [57,58,59,60,61]. Impairment of NK cells is associated with a reduced ability of the organism to target tumor cells and thus promotes cancer spread [62]. PDAC cells are able to develop mechanisms to inhibit NK cell function and thus protect themselves from the host immune system [63,64,65]. NK cells in the TME markedly downregulate activating receptors in response to immune evasion factors produced by tumor cells [66]. For example, tumor-derived lactate causes a downregulation of NCR1 (NKp46), therefore, inhibiting NK cell cytotoxicity [67]. Tumor cells can also secrete the protein IGHG1 (Igγ-1 chain C region), which attenuates the cytotoxic activity of NK cells by inhibiting ADCC [68]. He et al. discovered that PDAC cells actively consume vitamin B6, which NK cells need as a critical energy source for activation via glycogen metabolism [69]. Besides the direct influence of tumor cells, NK cells are indirectly affected by other immune cells and platelets found in the TME. For example, lactate increases the amount of MDSCs (myeloid-derived suppressor cells) in the tumor tissue and, therefore, leads to NK cell suppression indirectly [67]. It could be shown that natural killer T (NKT) cells have a beneficial effect on NK cell activity [70]. IL-15 (interleukin-15) produced by dendritic cells, monocytes, and macrophages increases NK cell cytotoxicity [71]. In contrast to that, platelets have a suppressive effect on NK cell function, as described in detail below [72,73].

#### 2.1.3. Clinical Implications in PDAC

Despite the limited functionality of NK cells as mentioned above, the amount of circulating NK cells in the peripheral blood of PDAC patients is a positive prognostic biomarker for resectable pancreatic cancer [74]. NK cells in the peripheral blood of PDAC patients display a reduced cytotoxic function and adopt a regulatory, IL-10-producing phenotype [66]. PDAC patients with high NK cell infiltration in the tumor displayed improved survival [69]. Interferon type I and II, produced by NK cells, are enriched in tumors with later recurrence [74]. As NK cells are able to kill tumor cells without prior sensitization, they have become a key focus of cell-mediated cancer immunotherapy. It could be shown that less differentiated tumor cells were more sensitive to NK cell-mediated killing, as higher differentiated tumors could re-increase their expression of MHC-I [75]. Highly differentiated cells were, in turn, more sensitive to chemotherapy. This suggests that a combination of surgical resection, chemotherapy, and immunotherapy might be a promising tool for the treatment of pancreatic cancer treatment. Especially after tumor resection and adjuvant treatment, NK cells play a crucial role in the control of recurrence [75]. Brooks et al. showed that the activation of CD8^+^ T cells and NK cells after surgery increased perioperative survival. Additionally, neoadjuvant anti-PD-1 and gemcitabine treatment, followed by tumor resection, and adjuvant anti-CD96 therapy, stimulated NK cell activation and led to significantly improved survival [76].

#### 2.1.4. Therapeutic Strategies

Various approaches exist to NK cell immunotherapy. One is to block NK cell MHC-I specific inhibitory receptors to increase NK cell effector function against tumor cells. This was successfully tested in mice with leukemia [77]. It is also possible to upregulate the expression of NKG2D, which is the strongest NK cell activator [52]. Another approach would be to isolate NK cells from the patient’s peripheral blood, followed by in vitro stimulation with IL-2 and/or co-culture with K562 tumor cells, resulting in proliferation and activation. NK cells are then released back into the patient’s bloodstream [52,78]. It is also possible to produce CAR-NK cells that bind to specific surface molecules of the tumor [52]. For example, CD70-targeting CAR NK cells were able to eliminate both PDAC cells and CAFs (cancer-associated fibroblasts) in vitro and in vivo when stimulated with IL-15 [79]. In conclusion, NK cells play a crucial role in tumor development, especially in the early stages, and are a promising target for immunotherapies.

### 2.2. Macrophages

#### 2.2.1. Key Physiological Functions

Macrophages play a wide role in tissue maintenance and pathological conditions, and their divergent role depends on several factors like origin, residence, and micro-environmental conditions [80]. Macrophages play a dynamic role in the immune system and are traditionally classified into two polarization states. While M1 macrophages are considered pro-inflammatory cells with anti-tumor properties, M2 macrophages have anti-inflammatory and pro-tumor properties [81]. Recent studies have revealed that macrophages can have mixed origins. In pancreatic cancer, both tissue-resident macrophages as well as inflammatory monocytes can be the source of TAMs (tumor-associated macrophages), exhibiting distinct functions depending on their source [82]. Macrophages are phenotypically highly plastic by nature, and polarization depends on external stimuli. In PDAC, macrophages are more prone to a pro-tumorigenic, or M2 polarization state. M2 macrophages can promote tumor angiogenesis, immune evasion, chemotherapy resistance, metabolic rewiring, and metastasis [37]. The phenotypic properties of macrophages highly depend on TME factors like hypoxia, cachexia, fibrosis, and immune-mediated secreted factors [80].

#### 2.2.2. Pathophysiology in Chronic Pancreatitis or PDAC

TAMs are the most abundant inflammatory cells in chronic pancreatitis and pancreatic cancer. TAMs are present and increase in number with disease progression from the early stage of PanIN to the metastatic stage of PDAC [83]. A detailed multi-institutional study of more than 300 patients by Väyrynen et al. reported that a higher density of macrophages in PDAC is associated with a worse prognosis and DFS (disease-free survival). While M2 macrophages were associated with worse prognosis and DFS, M1 macrophages did not show any specific correlation with prognosis [84]. However, macrophages might not only have disease-promoting functions but are also necessary for maintaining tissue homeostasis during pancreatic inflammation. Baer et al. identified a subset of TRMs (tissue-resident macrophages) showing high levels of LYVE1 and expression of genes important for tissue homeostasis. TRMs were necessary for tissue fibrosis and mediated a protective effect in cerulean-induced acute pancreatitis. In contrast, LYVE1+ TRMs supported pancreatitis-accelerated PDAC progression [85]. Another recent publication demonstrated the role of IL-1β^+^ TAMs in pancreatic cancer. This study highlighted that this subset of TAMs interacted with PDAC cells expressing an IL-1β response signature, leading to worse prognosis and survival of patients. Upon exposure to TNF (tumor necrosis factor) and PGE_2_ (prostaglandin E_2_) secreted by PDAC cells infiltrating monocytes differentiated to IL-1β^+^ TAMs and facilitated inflammatory reprogramming of cancer cells to potentiate the release of TNF, PGE_2_, and other factors for maintenance of an IL-1β^+^ TAM state. This positive feedback loop helped to mediate the inflammatory reprogramming leading to tumor-promoting inflammation, facilitating immune suppression and tissue repair in the tumor, and establishing the PGE_2_–IL-1β axis as a key driver of the inflammation-induced tumor progression [86]. Macrophages also play important roles in cachexia in pancreatic cancer. A current study highlighted the role of TWEAK (TNF-related weak inducer of apoptosis), a pro-inflammatory cytokine belonging to the TNF family, which led to muscle wasting. Specifically, tumor cells induced secretion of CCL2 (C-C motif chemokine ligand 2), leading to macrophage activation and subsequent TWEAK secretion by tumor cells via the CCL5/TRAF6 (TNF receptor-associated factor 6)/NF-κB signaling axis, which stimulated muscular atrophy [87]. Macrophage function and phenotypic switch largely depend on changes in their metabolic state [88,89]. M1 macrophage mediate their metabolism through glycolysis by increased glucose consumption, whilst M2 macrophages show an increase in oxidative phosphorylation [90]. A study performed by Zhang et al. using a co-culture model to understand the metabolic crosstalk between cancer cells and macrophages described the capability of PDAC cells to metabolically alter M1 macrophages to release the M2-specific cytokine IL-10. The results from the study suggested that the metabolic switch of M1 macrophages happens via direct interaction with PDAC cells, mediated by GARP (glycoprotein a repetitions predominant) and integrin αV/β8, and ultimately leads to TGF-β (transforming growth factor beta) secretion. Interestingly, selective reprogramming induced by PDAC cell interaction was only possible in M1 macrophages, whereas M2-like macrophages retained their phenotype upon tumor cell interaction. The same study highlighted that PDAC cells induced DNA methylation of genes related to glucose metabolism and oxidative phosphorylation (OXPHOS), including quinone oxidoreductase (NQO-1) and aldehyde dehydrogenase 1A3 (ALDH1A3), in M1 macrophages. This led to metabolic dysregulation. In M2 macrophages, these metabolism-related genes were upregulated [91]. Metabolic changes in macrophages might also influence chemoresistance. Halbrook et al. generated tumor-educated macrophages (TEMs) by growing bone marrow-derived macrophages in conditioned media from PDAC cells and performing metabolomics profiling. They identified an increased release of pyrimidine nucleosides and nucleobases in TEMs compared to control macrophages that impeded gemcitabine cytotoxic activity through molecular competition [92].

#### 2.2.3. Clinical Implications and Therapeutic Strategies

A deeper and better understanding of signaling pathways involved in tumor-associated macrophage recruitment and functional properties has led to the development of several macrophage-targeted therapies in various cancers. For example, trabectedin and its analog lurbinectedin selectively depleted monocytes and TAMs in patients and mice through the TNFRSF10 (tumor necrosis factor receptor superfamily member 10, also known as TRAIL)-dependent apoptosis pathway [93]. CD40, which is a key component of both the innate and adaptive immune system, remains an attractive target for cancer immunotherapy. CD40 receptor agonists are used to facilitate the production of TNF, reactive oxygen, and nitrogen species, which mediate the bactericidal and tumoricidal activity of macrophages. CD40 agonist administration caused an increased influx of CD4^+^ T cells into the TME. The safety of APX005M (CD40 agonistic antibody) was assessed in a phase I study in patients with metastatic pancreatic cancer. However, promising results demand further investigation in phase II/III clinical trials [94,95]. Altogether, this suggests that macrophages are an integral part of the pancreatic TME. The opportunity to understand TAM diversity at a single cell level and within a spatial context will enable a better characterization of pro- and anti-tumor behaviors of macrophages in PDAC [93].

### 2.3. Dendritic Cells

#### 2.3.1. Key Physiological Functions

Dendritic cells (DCs) are characterized as professional antigen-presenting cells [96] that bridge the innate and adaptive immune system [96]. DCs can differentiate in various ways to fulfill their tasks [96]. All DC subsets originate from HSCs (hematopoietic stem cells) within the bone marrow [97,98]. HSCs develop into CMPs (common myeloid progenitors) and CLPs (common lymphoid progenitors). CMPs generate imDCs (immature DCs) and monocytes, which can differentiate into cDCs (conventional DCs) or moDCs (monocyte-derived DCs) [98]. cDCs are further classified into cDC1s, which become interstitial DCs, and cDC2s, which differentiate into Langerhans cells in the epidermis [96,99,100,101]. The only DC subtype that develops from CLPs is the pDC (plasmacytoid DC) subtype, which can produce high amounts of type I IFN (interferon) to actively participate in antiviral immunity [100,102]. In PDAC, several DC subtypes can be affected. However, cDC1 dendritic cells are particularly important because of their ability to present antigens to T cells. Although this classification is simplified, recent studies have defined multiple new DC types with unique functional properties [103].

#### 2.3.2. Pathophysiology in PDAC

Disturbed maturation and a reduced number of DCs are fundamental problems in PDAC. The maturation of DCs can also be influenced by Reg3g (regenerating islet-derived protein 3 gamma) expression in PDAC cells. Reg3g promoted pancreatic cancer carcinogenesis via a STAT3 signaling pathway in a murine model of chronic pancreatitis [104]. Regarding an intratumoral immune response, Reg3g triggered an immunosuppressive microenvironment, including suppression of DC maturation, upregulation of EGFR (epidermal growth factor receptor) expression, and increase in Th2 cytokines in DCs while suppressing and inactivating CD8^+^ T cell responses [105]. Ochi et al. showed that MyD88 (myeloid differentiation primary response 88 innate immune signal transduction adaptor) inhibition exacerbated pancreatic inflammation and progression to invasive cancer [106]. TLR4 (toll-like-receptor 4) activation on DCs usually triggers either a MyD88-dependent or -independent pathway [107]. The MyD88-dependent pathway is the predominant one in DCs and leads to the production of pro-inflammatory cytokines, favoring Th1-differentiation. This, in turn, can activate anti-tumor immunity and suppress pancreatic tumor growth. Blocking the MyD88-dependent-pathway induces pancreatic antigen-restricted, Th2-deviated CD4^+^ T cells, leading to a transition from pancreatitis to pancreatic carcinoma [106]. A similar report by Lin and colleagues showed that cDC1 abundance and maturation declined progressively during pancreatic carcinogenesis. They remained in a semi-mature state associated with the upregulation of inflammation-related genes but impaired production of T-cell polarizing cytokines. Consequently, cDC1-mediated CD8^+^ T cell priming was compromised [108]. The TME, featuring hypoxia, dense stroma, a high presence of immunosuppressive cells like MDSCs and Tregs (regulatory T cells), and low pH due to the glycolytic metabolism of tumor cells can also affect DC function [109,110]. Elevated glycolytic activity in PDAC cells led to decreased DC infiltration and impaired antigen-presenting functions due to low glucose and high lactate concentrations, which were detrimental to DC mitochondrial and antigen-presenting capabilities [111]. Additionally, Treg infiltration suppressed the immunogenicity of DCs, contributing to a disturbed T cell activation [112]. Kenkel et al. described an immunosuppressive subset of DCs that promoted Treg expansion, thus leading to a higher risk of tumor metastasis [113].

#### 2.3.3. Clinical Implications in PDAC

Disturbed maturation and a reduced number of DCs are associated with poor prognosis and low survival rates in PDAC patients [114,115,116]. In addition, DCs play a crucial role in immunotherapy against various cancers, including PDAC [117,118,119,120]. PDAC patients exhibited decreased levels and enhanced apoptosis of cDCs and pDCs in comparison to control patients. Additionally, patients who have undergone tumor resection had lower DC counts even 12 weeks after surgery. Moreover, the authors found that high blood DC levels positively correlated with prolonged patient survival [120] and were usually associated with a better prognosis [121]. This is consistent with the data of Plesca et al., which showed that higher frequencies of tumor-infiltrating cDC1s and pDCs correlated with improved survival outcomes in PDAC patients, suggesting that the presence and functionality of DCs could serve as prognostic markers [122]. The same group claimed that the blood of PDAC patients accommodates more DCs with disturbed maturation in comparison to healthy patients. This includes both cDCs and pDCs. They showed an increased expression of CD83, CD40, B7H3 (also known as CD276), PD-L1 (also known as CD274), CCR6 (C-C chemokine receptor type 6), and CCR7 and a lowered expression of ICOSL (inducible T cell costimulator ligand), and DCIR, also known as CLEC4A (C-type lectin domain family 4 member A) [123]. Mechanistically, inflammatory factors, including elevated levels of PGE_2_ in PDAC patients’ plasma, were related to dysfunctional changes in DC phenotype [123]. Before pancreatic cancer development, particularly at the (IPMN) (intraductal papillary mucinous neoplasm) stage, increased secretion of CXCL17 and ICAM2 (intercellular adhesion molecule 2) led to the infiltration of cDCs, followed by T-cell-mediated cytolysis of tumor cells. Secretion of CXCL17 and ICAM2 was significantly decreased when IPMA progressed to pancreatic cancer, contributing to immune tolerance [124].

#### 2.3.4. Therapeutic Strategies

DCs are a fundamental component of immunotherapeutic approaches in numerous cancers, including PDAC [98,116,117]. One possibility is given by pulsing DCs with tumor-specific antigens, e.g., using cell lysates, KRAS G12D^1–23^ peptide, or alpha-enolase. As an example, in mice treated with alpha-enolase-pulsed DCs, T cell proliferation is stimulated. As a result, these cells secreted IFN-γ, which subsequently led to PDAC cell lysis [100,118]. Alternatively, DC vaccination might be combined with other treatments. Mahadevan et al. reported that using engineered cancer-specific cDC1s along with immunotherapy might be an approach for the treatment of pancreatitis-associated PDAC. They showed that ex vivo-engineered cDC1s stimulated with tumor antigens sensitized resistant PDAC tumors to immune checkpoint blockade [98]. A recent study showed that IL-2 promoted the expansion and intratumoral accretion of tumor-infiltrating DCs in PDAC [118]. Further approaches pursue the creation of a more favorable immune landscape to improve both DC infiltration and T cell activation and infiltration by inhibiting TNFR1 signaling or hypoxia-driven immune checkpoints [119,120]. Although DC-based therapy is promising in PDAC, further research is necessary to understand the fundamental biology and functionality of tumor-associated DCs to be able to improve or combine existing immunotherapeutic approaches.

### 2.4. Mast Cells

#### 2.4.1. Key Physiological Functions

Mast cells belong to the group of granulocytes and derive from myeloid progenitor cells [125]. During mast cell differentiation and maturation, mast cell progenitor cells typically migrate to areas of the body that are in direct contact with the environment, e.g., the skin, mucosa of the conjunctiva, and the respiratory and gastrointestinal tract [126]. Therefore, they are only found as immature precursor cells in the peripheral blood [101]. Fully differentiated mast cells are traditionally classified into three categories based on the dominant serine protease secreted: MC_T_ (tryptase-producing mast cells), MC_C_ (chymase-producing mast cells), and MC_TC,_ which produce both [127]. Further classification is based on the location of the mast cell: CMCs (connective tissue mast cells) can be distinguished from MMCs (mucosal mast cells) [128]. Mast cells play a vital role in allergic reactions, especially type 1 allergic reactions. Physiologically, mast cells bind IgE antibodies, which are produced by B-lymphocytes after first antigen contact. At the time of a second antigen contact, the antigen binds to IgE on the mast cell’s surface, causing its degranulation. Regulation of mast cells is mediated by the KIT receptor (CD117) [129]. Mast cell granules comprise, for instance, histamine, heparin, proteases (tryptase, chymase), and cytokines like interleukin (IL-1, -3, -4, -5, -8, -10), as well as PDGF (platelet-derived growth factor), TNF-α, TGF-β, and MMP9 (matrix metalloproteinase-9) [130].

#### 2.4.2. Pathophysiology and Clinical Implications in PDAC

The role of mast cells in cancer is multifaceted [131,132,133]. In some cancer types, mast cell infiltration of the TME is associated with better outcomes [134,135,136]. In other tumor entities, it has a negative impact on patient prognosis [137], e.g., for melanoma [138,139], breast cancer [140,141], and gastric cancer [142,143]. In PDAC, they are considered one of the tumor-promoting cell types [144], and mast cell infiltration is associated with a lower survival rate [145,146,147]. Tumor-infiltrating mast cells often accumulate at the edges of the tumor, which is also observed in PDAC [148,149]. Analyses of the mast cell count in pancreatic tissue from PDAC patients after curative partial pancreas resection showed a correlation between high mast cell count and TNM stage. Remarkably, only mast cell infiltration of the intratumoral border zone could be identified as an independent prognostic factor for overall survival, whereas survival did not correlate with the mast cell count in the intratumoral central zone or in the peritumoral zone [145]. A similar effect was also described for *K-ras^G12V^* mice, where mast cells accumulated at the tumor edge [146]. Therefore, they contributed significantly to the angiogenic switch during tumor progression [150,151]. Mast cells secrete several pro-angiogenic factors, such as VEGF (vascular endothelial growth factor), TNF-α, TGF-β, FGF-2 (fibroblast growth factor-2), and angiopoietin-1 [152,153,154,155,156]. Consistent with these findings, mast cell inhibition promoted the efficiency of anti-angiogenic therapy [157]. Other factors secreted by mast cells are IL-13 and tryptase, which directly induce the proliferation of pancreatic stellate cells (PSCs) [158,159]. On the other hand, tumor growth was suppressed significantly in mast cell-deficient mice [146,151]. In conclusion, mast cells infiltrate the TME of PDAC, promoting tumor progression by activating PSCs and facilitating angiogenesis.

#### 2.4.3. Therapeutic Strategies

Mast cells can be useful targets in cancer therapy. So far, three different approaches have evolved: mast cell inhibition, decreasing the mast cell count, or altering the effects of secreted mediators [160]. Although previous findings suggested that mast cell inhibition might be a promising approach, data for PDAC therapy are scarce [144]. Decreasing their chemotaxis by pharmacological inhibition of CXCR4 resulted in reduced mast cell migration in mice, and thus improved survival [158]. The tyrosine kinase inhibitor masitinib, which selectively targets the c-Kit receptor, increased overall survival in PDAC patients when combined with gemcitabine [161,162,163]. However, it is not yet known to what extent the effect of masitinib on mast cells contributes to these findings. Thus, further research is needed to elucidate the potential of mast cell inhibition in PDAC therapy.

### 2.5. Neutrophils

#### 2.5.1. Key Physiological Functions

Neutrophils are the most abundant innate immune cells in the blood and bone marrow [164,165]. They are the first cells being recruited to the site of inflammation and play a crucial role in host defense mechanisms, having antibacterial [166], antifungal [167], and antiviral [168] functions. Also, they perform phagocytosis of cell debris, an essential process for tissue regeneration and angiogenesis [169]. Some studies have proposed that two groups of tumor-associated neutrophils exist that differ according to their polarization state: tumor-suppressing N1 neutrophils and tumor-promoting N2 neutrophils [170].

#### 2.5.2. Pathophysiology and Clinical Implications in PDAC

Since neutrophils are considered major initiators of inflammation, they play a role in both acute and chronic pancreatitis as well as PDAC. Normal pancreatic tissue is not infiltrated by neutrophils, however, infiltration is significantly increased after cerulein-mediated tissue damage and release of PAF (platelet-activating factor) [171]. Neutrophils are recruited by the secretion of pro-inflammatory factors, such as TNF-α and IL-23. On the other hand, infiltrated neutrophils secrete a number of chemokines that further attract other immune cells of the innate and adaptive immune system [60]. In addition, the secretion of cytokines, including CXCL10 and CCL-21, can promote the migration of pancreatic cancer cells towards sensory neurons [172]. Moreover, neutrophil-derived elastase has been shown to mediate the dyshesion of tumor cells and degradation of E-cadherin, contributing to enhanced migration and invasion [173]. Another important function of neutrophils is the capability of forming NETs (neutrophil extracellular traps). NETs are large, web-like structures that contain neutrophil DNA as well as cytosolic and granule proteins [174] and are considered a defense mechanism for bacterial killing. Across several cancer types, NETs may promote tumor progression and metastasis (reviewed in [175]). Pancreatic cancer cells were able to induce NET formation [176] and the serum of PDAC patients has a higher NET-forming potential mediated by IL-17 [177]. In addition, tumor-derived TIMP-1 (tissue inhibitor of metalloproteinase-1) triggered NET formation upon interaction with its receptor CD63 and activation of ERK signaling. Inhibition of TIMP-1 or inhibition of NETosis prolonged survival in a murine PDAC model. Likewise, TIMP-1 levels correlated with local and systemic NET-formation in PDAC patients and negatively affected survival [178]. Furthermore, additional studies have shown that NETs induce migration, invasion, and EMT (epithelial–mesenchymal transition) of pancreatic cancer cells in an IL-1b/EGFR/ERK-dependent manner, which could potentially be utilized for therapy [179]. Interestingly, inhibition of PAD4 (protein arginine deiminase 4), which is critical for NET release, reduced NET formation and suppressed pancreatic tumor growth in a xenograft model [180]. NETs not only entrap pathogens but also bind platelets and contribute to cancer-associated thrombosis [181], which is associated with reduced survival in pancreatic cancer patients [182].

### 2.6. Myeloid-Derived Suppressor Cells (MDSCs)

#### 2.6.1. Key Physiological Functions

MDSCs are a heterogeneous cell population of pathologically activated neutrophils (granulocytic/polymorphonuclear MDSCs; PMN-MDSCs) and monocytes (monocytic MDSCs; M-MDSCs) that show strong immune suppressive capabilities and have been implicated in multiple malignancies [183]. While M-MDSCs are mainly responsible for T cell suppression in an antigen-specific and non-specific manner, associated with the production of nitric oxide and cytokines [184], PMN-MDSCs suppress T cells primarily in an antigen-specific manner [185]. The pathological activation and recruitment of MDSCs are mediated by a large variety of mostly pro-inflammatory cytokines, indicating the increasing importance of these cells in malignancies associated with chronic inflammation, such as chronic pancreatitis with associated pancreatic adenocarcinoma.

#### 2.6.2. Pathophysiology and Clinical Implications in PDAC

Monitoring infiltration of immune cells during disease progression in an LSL-*Kras^G12D/+^;p48^Cre^* mouse model showed an early influx of leukocytes into pancreatic tissue. Macrophage infiltration began very early in preinvasive disease, followed by an infiltration of MDSCs. MDSCs persisted after progression to PDAC and were found to be highly immunosuppressive in vitro [83]. Several studies have revealed that the homing of MDSCs to the pancreas in preinvasive and invasive diseases is mediated by cytokines directly produced within the pancreas. For example, PDAC cells were found to be able to produce high levels of GM-CSF (granulocyte-macrophage colony-stimulating factor) in mice. GM-CSF was capable of inducing splenocyte differentiation into MDSCs that suppressed T cell responses in vitro [186]. Likewise, human PDAC prominently expressed GM-CSF as well. Neutralizing GM-CSF blocked the recruitment of MDSCs and reduced tumor growth in mice that were dependent on CD8^+^ T cells [186]. In mice, data showed that the upregulation of GM-CSF was specifically dependent on oncogenic *Kras^G12D^* [187]. Furthermore, other factors including IL-1β, VEGF, CXCL1, and IL-6 can influence MDSC number in the tumor microenvironment, some of which are produced by PSCs. Mace et al. showed that PSCs secreted MDSC-promoting cytokines and chemokines and led to MDSC differentiation in a STAT3-dependent manner [188]. STAT3 signaling is suggested to be a crucial pathway regulating immunosuppressive activity in MDSCs. In head and neck squamous cell carcinoma patients, STAT3 signaling was responsible for arginase-I expression in MDSCs, which was crucial for MDSCs’ suppressive function [189]. Arginase-I has been shown to be a key driver of the immunosuppressive tumor microenvironment in PDAC [190]. In addition, the transformation of monocytes to M-MDSCs was dependent on STAT3 activation contributing to stemness and increased mesenchymal properties in pancreatic cancer [191]. Porembka et al. showed that C57BL/6 mice inoculated with Panc02 pancreatic cancer cells have increased numbers of MDSCs in their bone marrow and peripheral blood, which were recruited to the tumor site and were able to decrease T cell responses. Similar results were found in PDAC patients. Recruitment of MDSCs and tumor growth could be inhibited by zoledronic acid treatment in vivo [192]. Unfortunately, translating these findings to PDAC patients was not successful as zoledronic acid did not impact overall or progression-free survival [193]. Besides inhibition of CD8^+^ T cell function directly, MDSCs can also stimulate the expansion of immunosuppressive Tregs via TGF-β and IFN-γ [194,195], which contributes to impaired CD8^+^ T cell function as well. In addition, the influence on NK cells as well as macrophages can also attenuate anti-tumor immunity [196,197,198].

#### 2.6.3. Therapeutic Strategies

As MDSCs are key drivers of immune suppression in pancreatic cancer, targeting MDSCs might represent a potential tool to promote anti-tumor response. Interestingly, gemcitabine is known to decrease the quantity of MDSCs, thereby promoting antitumor response [199]. Another approach includes targeting mediators that promote their proliferation and recruitment, e.g., GM-CSF, as mentioned above. In addition, inhibitors of the JAK2/STAT3 signaling pathway might limit MDSC stimulation and infiltration. This was tested in immunocompetent PDAC mouse models, where the authors used CXCR4-modified Claudin 18.2-directed CAR-T cells that suppressed recruitment of MDSCs via a STAT3/NF-κB/SDF-1α axis. CXCR4 not only led to a better infiltration of CAR-T cells into tumor sites but also increased the efficacy of CAR-T cell therapy by reducing the production of STAT3-mediated cytokines [200].

While current and novel therapeutic approaches might reduce MDSC function and infiltration, it is known that chemotherapy in PDAC leads to the release of inflammatory factors, including GM-CSF, that contribute to increased numbers of intratumoral MDSCs [201,202]. In conclusion, MDSCs play a crucial role in shaping the tumor immune microenvironment in PDAC, thereby affecting initiation, disease progression, and therapy response. Understanding their function and regulation might further help to design innovative therapeutic strategies that impact the PDAC TME.

## 3. Platelets as Regulators of Tumor Growth and Immunity

Platelets are increasingly recognized as active contributors to tumor growth, immunity, and immune evasion beyond their traditional role in hemostasis. Interaction with immune or cancer cells happens either directly via receptor–ligand interactions or indirectly via the secretion of cytokines or growth factors. Thus, platelets are positioned at the intersection of inflammation, tumor immunity, and cancer progression.

### 3.1. Key Physiological Functions

Platelets or thrombocytes are blood components that, although not categorized as immune cells, can have considerable roles in inflammation and immune response. Megakaryocytes are the precursors of platelets and reside in the bone marrow, where platelet formation primarily occurs [203]. After their release from megakaryocytes into the blood vessels, platelets have a lifespan of 5–7 days [204]. Being highly abundant in circulation, platelets play a key role in regulating hemostasis. They are able to sense damage to blood vessels within seconds as they recognize glycosylated structures of the extracellular matrix, like collagen, von Willebrand factor, laminin, or fibronectin, that become exposed when the vessel is injured. Subsequently, platelets become activated [205]. In the past years, accumulating evidence suggests that platelets become activated by tumor cells as well and that they are not merely bystander cells but play active roles in several steps of tumorigenesis [206]. For example, platelets were shown to regulate tumor growth and therapy response [207,208]. Moreover, platelets interact with circulating tumor cells and protect them from anoikis, thereby promoting metastasis formation [209]. In addition, several studies have highlighted platelets as orchestrators of immunity and regulators of the cancer immune microenvironment with potential roles in immune evasion. It is well known that platelets store and release chemokines and cytokines upon activation that can regulate the recruitment, activation, and differentiation of immune cells [210]. In addition, platelets interact with immune cells of innate and adaptive immunity via receptor–ligand interactions, e.g., PSGL-1 (P-selectin-P-selectin glycoprotein ligand-1 interaction) that mediates rolling and adhesion of neutrophils [211]. Similarly to innate immune cells, they express PRRs (pattern recognition receptors) like TLRs, NLRs (NOD-like receptors), and CLR (C-type lectin receptors) on their surface [212] that recognize damage- or pathogen-associated molecular patterns, and they communicate with or present antigens to T cells [213].

### 3.2. Impact of Platelets on Immune Cells of the Innate Immune System

**Figure 4:**
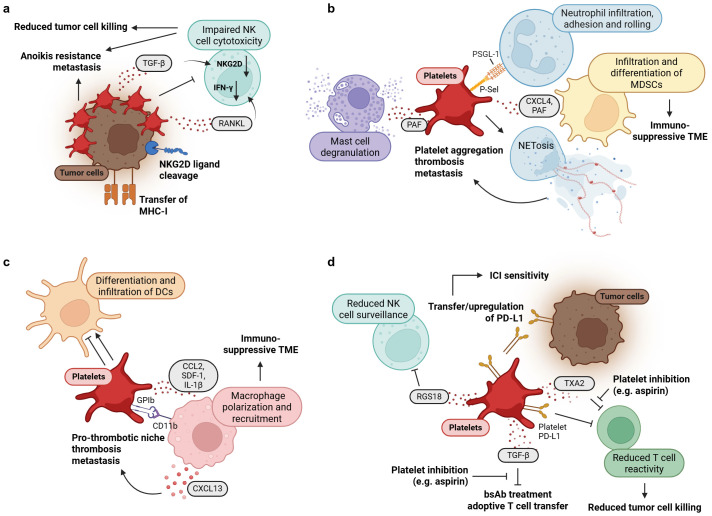
Platelet collaboration with innate immune cells, their role in cancer, and in immune evasion. (**a**) Tumor cell coating protects CTCs from anoikis, supports their dissemination, and shields them from NK cell cytotoxicity by secreting TGF-β or RANKL (receptor activator of nF-κB ligand). This reduces NKG2D expression or IFN-γ secretion. In addition, platelets mediate cleavage of NKG2D ligands or transfer MHC class I molecules onto the tumor cell surface. (**b**) Platelets mediate infiltration and differentiation of MDSCs, and ultimately support an immunosuppressive TME, via CXCL4 and PAF (platelet-activating factor), adhesion and rolling of neutrophils by receptor–ligand interactions, e.g., P-Sel (P-Selectin)-PSGL-1 (P-selectin glycoprotein ligand-1) among others, and induce the formation of neutrophil extracellular traps (NETosis). NET formation contributes to platelet aggregation, thrombosis and metastasis. Furthermore, platelets can induce the degranulation of perivascular mast cells via PAF. (**c**) Platelets induce macrophage polarization and recruitment via direct and indirect mechanisms and regulate differentiation and infiltration of DCs. Polarization to an M2-phenotype in tumors drives an immunosuppressive TME. Moreover, macrophages can induce a pro-thrombotic niche via CXCL13 secretion that stimulates thrombosis and metastasis. (**d**) Platelets may transfer to or upregulate PD-L1 expression in tumor cells that predicts ICI treatment sensitivity. In addition, platelet PD-L1, as well as, secretion of TXA2 (thromboxane A2) or TGF-β, reduces T cell reactivity and efficacy of T cell-based immunotherapy that can be alleviated by platelet inhibitory agents, including aspirin. Immune evasion from NK cell surveillance is induced by platelet RGS18 (regulator of G protein signaling 18). The figure was created using BioRender.com.

In several murine models of inflammation, platelets have been key in regulating the extravasation of immune cells [214,215]. When platelet numbers were low, host defense in bacterial pneumonia was impaired [216,217]. Likewise, in cancer, immune cell infiltration and activity can be regulated by platelets. This is the result of direct platelet-immune cell interactions or cytokine secretion after platelet activation (Figure 4a–c). Subsequently, this can impact tumor growth and metastasis.

It has long been recognized that NK cell-mediated tumor lysis is impaired by platelets forming a platelet coat around circulating tumor cells [218]. Mechanistically, it has been shown that platelet coating led to a transfer of MHC class I molecules onto the tumor cell surface that disrupted NK cell recognition and impaired cytotoxicity [73]. Alternatively, platelet coating reduced surface expression of NKG2D ligands, in particular, MHC class I chain-related sequence A (MICA) and MICB, on tumor cells and thereby diminished NKG2D-dependent lysis of tumor cells [219]. NKG2D expression can also be downregulated by platelet-derived TGF-β [72]. Moreover, platelet RANKL (receptor activator of nF-κB ligand) reduced IFN-γ production by NK cells and inhibited NK cell cytotoxicity [220].

Platelets can also regulate the differentiation of innate immune cells. This has been shown for DCs, where platelets reduced DC differentiation and altered production of proinflammatory and immunoregulatory cytokines [221]. In contrast to these findings, another study reported that platelets enhanced DC maturation, infiltration, and their capacity to stimulate lymphocyte proliferation [222]. Similarly, the polarisation of macrophages can be regulated by platelet interaction. Here, studies show that platelets can promote both a polarisation towards an M1- and an M2-phenotype, likely depending on the model used. In a murine sepsis model, platelets promoted macrophage polarization towards a pro-inflammatory M1 phenotype in a cell contact-dependent manner involving the glycoprotein Ib (GPIb)-CD11b axis [223]. In cancer, platelet aggregation increased hematogenous metastasis formation by triggering macrophage recruitment and promoting an M2 phenotype [224]. Other results showed that platelet-derived CXCL4 induced a unique phenotype in macrophages, showing characteristics of both M1 and M2 macrophages. The authors called these M4-macrophages [225]. A strong functional interplay between macrophages and platelets is also demonstrated for cancer-associated thrombosis driven by a pro-thrombotic niche that was induced by CXCL13-reprogrammed macrophages. This promoted metastasis across multiple cancer types including a murine PDAC model [226].

Due to their rapid response after vessel injury, platelets are ideally positioned between the damaged endothelium and infiltrating neutrophils. Indeed, direct interactions of platelets and neutrophils seem to be crucial for inflammatory processes, NETosis, thrombosis, and cancer. Interaction between neutrophils and platelets is mediated via multiple receptor–ligand pairs, including P-Selectin-PSGL1, ICAM2-LFA1 (lymphocyte function-associated antigen 1), JAM-3 (junctional adhesion molecule 3), Mac-1 (macrophage-1 antigen), and CD40-CD40L [211,227,228,229]. Platelets foster transmigration and extravasation of neutrophils after vessel injury, which is required for subsequent effective immune responses and tissue repair [230]. On the other hand, neutrophils trigger platelet activation and thrombosis during systemic infections, which suppresses pathogen dissemination [231]. It has been shown that neutrophil recruitment in the early phase of thrombus formation is indispensable for deep venous thrombosis (DVT), partly driven by tissue factor secretion. In turn, DVT propagation is supported by platelets via stimulation of NETs [232]. NET formation can be induced by cytokines secreted via tumor cells [233] or is dependent on platelet proteins, receptors, or adhesion molecules [234,235,236].

The impact of platelets on MDSCs is relevant for local immunosuppression. It has been shown that platelets increase the differentiation of monocytes into MDSCs, which inhibit CD8^+^ T cell function [237]. A similar report by Servais et al. showed that platelets enhanced immunosuppression, MDSC infiltration into tumors and MDSC-mediated inhibition of T cell proliferation [238].

While the interactions between platelets and above mentioned innate immune cells are well characterized, the significance of platelet–mast cell crosstalk remains largely unexplored. It has been shown that platelets trigger perivascular mast cell degranulation via PAF [239]. In addition, platelets and mast cells might work together to promote the recruitment of eosinophils during fungal infections [240]. However, their interaction in the context of cancer has not been studied so far.

In summary, platelets play a pivotal role in modulating innate immune responses during cancer and inflammation. Platelet–immune cell interactions can trigger pro-inflammatory and pro-thrombotic pathways that link hemostasis, immunity, and oncogenesis.

### 3.3. Role of Platelets in Immune Evasion

A meta-analysis of 12 studies with 1340 cancer patients showed that the platelet–lymphocyte ratio (PLR) is a negative predictor of survival under immune checkpoint inhibitor therapy (ICI) [241]. This raised the question of whether PLR is a simple biomarker or if platelets, either alone or through interactions with immune cells in the tumor microenvironment, actively influence response to ICI, immune evasion, and disease outcome. Indeed, it has been shown that platelets can increase PD-L1 expression in cancer cells [242,243], a well-accepted and long-standing marker of immune evasion in several types of cancer [244]. Similarly, a report showed that platelet PD-L1 was able to protect PD-L1 negative tumor cells from elimination by T cells [245]. Platelets are also capable of transferring PD-L1 to tumor cells. This was shown for lung cancer cells and was dependent on fibronectin 1, integrin α5β1, and GPIbα. Platelet PD-L1 inhibited CD4^+^ and CD8^+^ T cells and predicted response towards ICI [246]. Other T cell-based immunotherapies, e.g., T cell-recruiting bsAb (bispecific antibody) treatment, can be modulated by platelets as well. A study showed that platelets significantly reduced bsAb-mediated CD4^+^ and CD8^+^ T cell reactivity mediated by platelet TGF-β, providing a rationale for modulation of platelet function to reinforce the effectiveness of bsAb treatment [247]. Platelet effects are not limited to bsAb treatment but have been shown to be useful for adoptive T cell transfer as well. Platelet TGF-β reduced the efficacy of adoptive T cell transfer in murine melanoma, which could be mitigated by platelet inhibitory drugs, including aspirin and clopidogrel [248]. In general, platelet inhibitory drugs might hold great promise to overcome immune evasion. In a murine ovarian cancer model, aspirin in combination with adoptive T cell transfer led to a markedly increased infiltration of tumor-reactive CD8^+^ T cells [249]. Similar results have been demonstrated recently, showing that aspirin prevented metastasis formation by releasing T cells from suppression of platelet thromboxane A2 [250]. NK cell-based treatments may also be affected by platelets, as numerous in vitro and vitro studies have demonstrated that platelets suppress NK cell cytotoxic activity, as highlighted above. A recent study in PDAC demonstrated that immune evasion of CTCs from NK cell surveillance, engaging the HLA-E:CD94-NKG2A immune checkpoint, is promoted by platelet-derived RGS18 (regulator of G protein signaling 18). RGS18 significantly increased hepatic metastasis, which was inhibited by the blockade of NKG2A or the knockdown of HLA-E [251].

Overall, these findings underscore the role of platelets as key contributors to immune evasion and the effectiveness of immunotherapy (summarized in Figure 4d), thereby supporting the rationale for incorporating anti-platelet therapy into certain treatment strategies.

### 3.4. Pathophysiology and Clinical Implications in PDAC

Pancreatic cancer cells were shown to induce platelet aggregation and activation [252]. This is associated with a release of bioactive molecules from platelet granules. Moreover, pancreatic cancer cell-derived tissue factor (TF) can lead to activation of coagulation and thrombus formation [253,254]. In fact, pancreatic cancer patients are especially prone to venous thrombosis [255], and paraneoplastic thrombocytosis is associated with worse survival in PDAC [256,257]. In line with this, the relative risk for the subsequent diagnosis of an occult pancreatic carcinoma after the occurrence of a thromboembolic event is six-fold higher [258]. In addition, high platelet-to-albumin ratios as well as high platelet-lymphocyte ratios are negative predictors of PDAC patient survival [259,260]. This suggests that platelets might be used as diagnostic and prognostic biomarkers. Indeed, the use of the RNA content of tumor-educated platelets as a source for liquid biopsy has been proposed [261]. Platelets might also be important for pancreatitis and PDAC development. Upon platelet activation, TGF-β can be released from platelet granules [262], which is a critical immunomodulatory cytokine and highly abundant in chronic pancreatitis and pancreatic cancer (reviewed in [263]). Activation of the TGF-β signaling pathway was found to be a driving force for fibrosis in chronic pancreatitis, acinar-to-ductal metaplasia, and pancreatic tumor initiation [264,265]. Furthermore, platelets actively release IL-1β [266,267], which is associated with acute and chronic pancreatitis as well as PDAC promotion [268,269]. Indeed, pannexin 1 led to increased IL-1β secretion in CD41^+^/CD62^+^ platelets that promoted migration and invasion of PDAC cells in vitro. Inhibition of pannexin 1 using a small molecule inhibitor proved its relevance also in vivo by inhibition of primary tumor growth, metastasis, and survival [270]. In addition, analyzing TCGA pancreatic cancer transcriptomics data showed an inverse correlation between platelet and CD8^+^ T cell RNA signatures but a positive correlation with MDSC RNA signatures [237], indicating that platelets are crucial for regulating the infiltration of respective immune cells into pancreatic tumor tissue. Likewise, the expression of platelet-related genes in PDAC has been associated with immune cell infiltration and immune checkpoint expression [271]. Moreover, evasion of human PDAC CTCs from NK cell surveillance has been mediated by platelets [251].

Although several indirect associations exist, the question of whether platelets directly influence early pancreatic carcinogenesis and PDAC progression still requires more detailed investigations and is the subject of ongoing research.

### 3.5. Therapeutic Strategies

Platelets might not only be important for cancer development but also for cancer treatment. For example, inhibiting platelet activity by combining aspirin and the P2Y12 inhibitor clopidogrel was able to reduce progression to hepatocellular carcinoma [272]. Aspirin may hold global significance, especially regarding the prevention of pancreatic cancer [273]. In addition, the homing capabilities of platelets have been harnessed in systems where platelet mimics were loaded with a cytotoxic payload to restrict the localization of the drug to the tumor site [274,275,276]. The most interesting application of this methodology includes perforin/granzyme-loaded platelets that are able to kill tumor cells upon activation and subsequent alpha granule release directly at the tumor site [277]. Platelets and platelet inhibitory drugs might also regulate the efficacy of immunotherapy, as highlighted above [246,247,248,249,250]. Very recently, combining immunotherapy with antithrombotic therapy has been reported to improve survival of advanced melanoma patients [278]. Although PDAC is an immunologically cold tumor with low T cell infiltration and restricted efficacy of immunotherapeutic agents, adding platelet-based therapies might be a novel approach to handling this deadly disease. However, whether such a therapeutic strategy is effective in PDAC remains to be determined.

## 4. Future Directions and Perspectives

Intricate crosstalk exists between the innate immune system, tumor cells, and platelets that drive intratumoral inflammation, fibrosis, tumor growth, metastasis, and therapy response. However, innate immune cells and platelets also critically shape responses of the adaptive immune system. In particular, the role of platelets in regulating T cell responses and immune evasion was described in detail above. In addition, dysregulation of the innate immune system can profoundly impair adaptive immune responses, particularly in the context of cancer. Uptake and presentation of tumor antigens is a prerequisite for subsequent priming and activation of naϊve T cells. Here, dendritic cells play a central role in generating specific T cell-mediated antitumor responses (see also Figure 3) to control tumor growth and dissemination [279]. However, the phenotype and function of DCs are altered by inflammatory mediators generated in the TME, which impairs their antigen-presenting capacity [280]. In addition, NK cells influence adaptive immunity and can both stimulate and inhibit T cell responses (see also Figure 2). Regulation of T cell responses can be direct or indirect. Direct mechanisms include the secretion of cytokines, e.g., IFN-γ or IL-10 that mediate T cell differentiation or T cell inhibition, respectively. Alternatively, they are able to kill CD4^+^ and CD8^+^ T cells in an NKG2D- and perforin-dependent manner. Effects on T cells can also be indirect via governing DC maturation and elimination or antigen cross-presentation [281]. The recruitment of immunosuppressive cells, including MDSCs and TAMs, is an additional mechanism to reduce anti-tumor T cell responses and the efficacy of immunotherapy. Similar to NK cells, both direct and indirect regulatory mechanisms exist. They inhibit T cell functions through the production of reactive oxygen species, arginase-1, IL-10, and nitric oxide. In addition, they can restrain the intratumoral localization of cytotoxic T cells while promoting the recruitment of regulatory T cells. Moreover, their presence in the tumor microenvironment can limit the effectiveness of ICI [80,183]. In summary, an effectively regulated innate immune system is crucial for orchestrating robust and functional adaptive immune responses that can also be limited by platelets. This interplay is particularly relevant for PDAC, where a dominant immunosuppressive environment hinders effective antitumor immunity. Therefore, targeting innate immune cells or platelets, alongside T cell-based therapies such as immune checkpoint blockade, represents a promising therapeutic strategy. Indeed, several currently ongoing and recently completed approaches are used in PDAC that target these cellular compartments and are summarized in Table 1.

Furthermore, emerging technologies such as single-cell RNA sequencing (scRNA-seq), spatial transcriptomics, and high-dimensional immune profiling are transforming our understanding of the tumor microenvironment. This is critical, particularly in PDAC, which is characterized by extensive cellular heterogeneity and a dense stromal compartment. This cellular heterogeneity could be uncovered using single-cell transcriptomics, e.g., in primary PDAC, metastatic lesions, as well as PDAC-derived organoids [282,283]. To further differentiate the cellular subtypes, scRNA-seq enabled the high-resolution characterization of rare or previously unrecognized subpopulations of immune cells [284,285,286], fibroblasts [287,288], and malignant epithelial cells. Spatial transcriptomics complements scRNA-seq by preserving the spatial architecture of the tumor, allowing researchers to map specific gene expression patterns to discrete regions within the tumor and stroma. This is crucial for understanding how cell–cell interactions and gradients of signaling molecules influence tumor progression, immune evasion or metastasis. Immune profiling, including high-dimensional flow cytometry and multiplexed imaging, adds another layer by quantifying immune cell subsets, their activation states, and interactions with other cell types such as platelets, cancer-associated fibroblasts, and endothelial cells [289]. Together, these approaches offer a powerful toolkit for dissecting the immunological landscape of PDAC, identifying novel biomarkers and causes for interindividual differences in drug responses as well as developing strategies for precision immunotherapy [36,290]. Integrating these insights with functional and translational studies will be pivotal for identifying new therapeutic targets and guiding the development of more effective, personalized immunotherapies for PDAC.

## 5. Conclusions

Chronic pancreatitis is a global health problem and an important risk factor for PDAC, which remains one of the most lethal malignancies worldwide. The innate immune system and platelets play multifaceted and often context-dependent roles in the pathogenesis and progression of chronic pancreatitis and PDAC. Persistent inflammation, partly driven by innate immune cells and platelets, contributes to tissue remodeling and fibrosis, setting the stage for malignant transformation. In PDAC, these same cells often adopt tumor-promoting phenotypes that support immune evasion, angiogenesis, and metastasis. Clinically, the complex crosstalk between innate immune cells, platelets, and tumor cells underscores the limitations of monotherapies in PDAC. A more effective approach will likely involve combination strategies that target both the tumor and its immunosuppressive microenvironment. Integrating innate immune modulators with T cell-based therapies holds promise for overcoming resistance and improving patient outcomes. In conclusion, understanding the diverse roles of innate immune cells and platelets in pancreatic inflammation and malignancy offers critical insights into disease mechanisms and reveals novel opportunities for therapeutic intervention in PDAC. Continued research into the dynamic interactions within the pancreatic tumor microenvironment is essential to translate these insights into effective clinical strategies.

## Figures and Tables

**Table 1:** Summary of ongoing and recently completed clinical trials targeting innate immune cells or platelets in PDAC. 5-FU: 5-Fluorouracil; ACT: Adoptive cellular therapy; CAR: chimeric antigen receptor; CCR: CC motif chemokine receptors; CD: Cluster of differentiation; CSF1R: Colony-stimulating factor 1 receptor; mTOR: Mammalian target of rapamycin; PI3K: Phosphatidylinositol 3-kinase; SIRPα: Signal-regulatory protein α; STING: Stimulator of interferon genes; CXCR: CXC motif chemokine receptor; TKI: tyrosine kinase inhibitor; ILRAP: Interleukin-1 receptor accessory protein.

**NK Cells**			
**Compound**	**Clinical Study Phase**	**Trial Number**	**Combination Drugs/Treatment**
CAR-NK Cells (CL-NK-001)	Phase 1	NCT06816823	-
Allogeneic Magicell-NK infusion	Phases 1/2	NCT06730009	SLOG chemotherapy
CAR-T/CAR-NK cells	Phase 1	NCT06572956	-
Chemotherapy Sequential NKG2D CAR-NK Cell	Phase 1	NCT06503497	Second-line chemotherapy (not specified)
Intratumoral Injection and intravenous infusion of NKG2D CAR-NK cells	Phase 1	NCT06478459	-
CAR-NK cells (CB CAR-NK182) targeting Claudin18.2	Phase 1	NCT06464965	-
TROP2-CAR/IL15-transduced CB-NK cells	Phases 1/2	NCT05922930	Cyclophosphamide, Fludarabine
NK cell infusion	Phases 1/2	NCT02718859	Irreversible electroporation (IRE)
Cytokine-induced Killer Cells	Phases 1/2	NCT01868490	-
Dendritic cell-activated Cytokine-induced killer treatment (DC-CIK)	Phases 1/2	NCT01781520	S-1
Interleukin-2	Phase 2	NCT05810792	Histamine Dihydrochloride (HDC)
FT500	Phase 1	NCT03841110	Cyclophosphamide, Fludarabine, Nivolumab, Pembrolizumab, Atezolizumab, IL-2
FATE-NK100	Phase 1	NCT03319459	Cetuximab, Trastuzumab
**Macrophages**			
**Compound**	**Preclinical/clinical study**	**Trial Number**	**Combination drug**
BMS-813160 (CCR2/CCR5 antagonist)	Phases 1/2	NCT03184870	Nivolumab, nab-paclitaxel, gemcitabine, 5-FU, leucovorin, irinotecan
BMS-813160	Phases 1/2	NCT03767582	Nivolumab, GVAX
TAK-500 (CCR2 and STING agonist)	Phase 1	NCT05070247	Pembrolizumab
PLX3397 (CSF1R inhibitors)	Phase 1	NCT02777710	Durvalumab
LY3022855 (anti-CSF1R antibody)	Phase 1	NCT03153410	Cyclophosphamide, GVAX, pembrolizumab
Cabiralizumab (anti-CSF1R antibody)	Phase 1	NCT02526017	Nivolumab
Lurbinectedin	Phase 2	NCT05229588	-
BI 754,091 (anti-CD47/SIRPα antibody)	Phase 1	NCT04752215	Ezabenlimab
CP-870, 893 (CD40 agonist antibody)	Phase 1	NCT01456585	Gemcitabine
APX005M (CD40 agonist antibody)	Phase 1	NCT02600949	Pembrolizumab, sotigalimab
APX005M	Phases 1/2	NCT05419479	Zimberelimab, domvanalimab
RO7009789 (CD40 agonist antibody)	Phase 1	NCT02588443	Nab-paclitaxel, gemcitabine
Gedatolisib (PI3K/mTOR inhibitor)	Phase 1	NCT03065062	Palbociclib
Metformin	Phase 1	NCT02336087	Gemcitabine, paclitaxel albumin-stabilized nanoparticle formulation
Metformin	Phase 2	NCT01666730	Oxaliplatin, leucovorin calcium, fluorouracil
Metformin	Phase 2	NCT04033107	Vitamin C
CT-0508 (CAR macrophages)	Phase 1	NCT04660929	Pembrolizumab
**Dendritic cells**			
**Compound**	**Preclinical/clinical study**	**Trial Number**	**Combination drug**
Metformin	Phase 2	PREOPANC trial EudraCT: 2012-003181-40	-
Toripalimab	Phases 1b/2	ChiCTR2000032293	chemotherapy
Ibrutinib	Phase 1/2	NCT02562898	Paclitaxel, gemcitabine
Wilms’ tumor 1 (WT1) peptides	Phase 2	jRCTc030190195	multiagent chemotherapy
**Mast cells**			
**Compound**	**Preclinical/clinical study**	**Trial Number**	**Combination drug**
Masitinib (TKI targeting KIT)	Phase 3	NCT00789633	Gemcitabine
**Neutrophils**			
**Compound**	**Preclinical/clinical study**	**Trial Number**	**Combination drug**
Nadunolimab (IL1RAP inhibition)	Phases 1/2	NCT03267316	Cisplatin, gemcitabine, nab-paclitaxel, carboplatin, pemetrexed
**MDSCs**			
**Compound**	**Preclinical/clinical study**	**Trial Number**	**Combination drug**
Entinostat	Phase 2	NCT03250273	Nivolumab
SX-682 (CXCR1/2 Inhibitor)	Phase 1	NCT04477343	Nivolumab
**Platelets**			
**Compound**	**Preclinical/clinical study**	**Trial Number**	**Combination drug**
Avatrombopag	Phase 1	NCT06182072	Gemcitabine, nab-Paclitaxel
